# Trends in Subdural Hemorrhage-Related Mortality and Its Geodemographic Disparities Among Older Adults in the United States

**DOI:** 10.1227/neuprac.0000000000000154

**Published:** 2025-08-19

**Authors:** Martin G. McCandless, Anand A. Dharia, Jonathan Swiastyn, Siddharth Shah, Paul J. Camarata

**Affiliations:** Department of Neurological Surgery, University of Kansas Medical Center, Kansas City, Kansas, USA

**Keywords:** Epidemiology, Subdural hemorrhage, Elderly, Mortality

## Abstract

**BACKGROUND AND OBJECTIVES:**

Subdural hemorrhage (SDH) is a common and potentially devastating intracranial injury routinely treated in neurosurgical practice. There are limited data regarding the mortality rates associated with SDH in older adults. Quantifying SDH-related mortality is crucial, especially considering the potential financial burden from ongoing medical and rehabilitative care associated with SDH morbidity and mortality in the elderly.

**METHODS:**

Adults aged 65 years or older in the United States whose deaths attributed to SDH occurred between 1999 and 2020 were extracted from the Centers for Disease Control and Prevention's Wide-ranging Online Data for Epidemiological Research database. Ethnicity and race were divided into exclusive categories as Hispanic or Latino, non-Hispanic Black or African American, or non-Hispanic White. SDH-related age-adjusted mortality rate (AAMR) per 100 000 persons was determined by standardizing the crude mortality rate to the 2000 US population. Overall population and subgroup segmented regression analyses were performed using Joinpoint Regression Program.

**RESULTS:**

Total of 203 295 SDH-related deaths occurred in older adults between 1999 and 2020 (AAMR 22.1 per 100 000 individuals). Of the total deaths, 110 684 (54.4%) were men, 92 611 (45.6%) women, 170 953 (84.1%) non-Hispanic White, 14 654 (7.2%) non-Hispanic Black or African American, and 9656 (4.7%) Hispanic or Latino. Overall AAMR increased from 17.4 in 1999 to 24.6 in 2020 with an average annual percent change of 1.3%; 95% CI (0.9, 1.8). The AAMR had an increase from 1999 to 2005 (annual percent change, 3.4%; 95% CI [2.0, 4.9]) followed by a slower increase from 2005 to 2020 (annual percent change, 0.5%; 95% CI [2.0, 4.9]).

**CONCLUSION:**

There is an alarming rise in overall SDH-related mortality among the elderly population in the United States, and notable disparities in SDH outcomes across various demographic and geographic sectors continue to exist. Future health interventions aimed at SDH need to address the disparities as highlighted in this study.

**ABBREVIATIONS:**
AAMR: age-adjusted mortality rate per 100 000 people
APC: annual percent change
CMR: crude mortality rate
MMAE: middle meningeal artery embolization
SDH: subdural hemorrhage.

Subdural hemorrhage (SDH) is a common and potentially devastating intracranial injury routinely treated in neurosurgical practice. SDH may be chronic or subacute after minor or no reported trauma but often present acutely after severe head injury. Risk factors of SDH include age, male sex, and anti-platelet or anticoagulant use.^1,2^ Current estimates of chronic SDH incidence range from 1.7 to 20.6 per 100 000 individuals, but the incidence is higher in the elderly population.^3^ Overall incidence of SDH is increasing,^4-6^ and some researchers have estimated the annual volume in the United States will increase by 78.3% by 2040.^7^

The incidence of SDH increases with age.^3,4,7^ Multiple factors contribute to this elevated incidence including increased fall risk, cerebral atrophy, anticoagulation/antiplatelet use, and other comorbid conditions common in elderly populations.^1,4,8-10^ Consequently, an expanding elderly population is frequently cited as contributing to the increasing incidence of both acute and chronic SDH.^3,4,7^ SDH also continues to represent a significant financial burden on patients.^4^ One national study found the average cost of SDH treatment in the United States increased 67% over a 9-year period.^8^

Given the current aging population and epidemiological trends, SDH incidence will likely continue to significantly increase in the United States.^7^ Despite the significant financial and clinical impact of SDH, there is a paucity of literature describing trends in SDH-related mortality. A robust epidemiological understanding of SDH will aid in developing targeted health measures to combat the significant impact of this increasingly prevalent neurological disease. In this study, we investigate trends in SDH-related mortality among adults aged 65 years or older using data from the Centers for Disease Control and Prevention's Wide-ranging Online Data for Epidemiological Research (CDC WONDER) database. In-depth investigation of SDH-related mortality trends will contribute to the current body of SDH epidemiological literature. This knowledge will also support further development of clinical guidelines and targeted programs to better attenuate the impact of SDH-related mortality.

## METHODS

This study analyzed the number of deaths attributed to SDH in adults aged 65 years or older in the United States between 1999 and 2020. The data were extracted from the CDC WONDER database specifically utilizing the Multiple Cause-of-Death Public Use death certificates provided by the National Center for Health Statistics.^11^ SDH was identified as either a contributing or underlying cause of death using the International Statistical Classifications of Diseases, 10th Revision, Clinical Modification codes for nontraumatic SDH (I62.0) and traumatic SDH (S06.5).^12^ Annual SDH-related deaths and corresponding population sizes were obtained and stratified by diagnosis, sex, race and ethnicity, urbanization, and state. This study did not require institutional review board approval or patient consent because of the deidentified nature of the publicly available data. Analysis was conducted on February 2024.

Race and ethnicity were reported on death certificates following the standards from the US Office of Management and Budget.^13^ Participants were categorized into mutually exclusive groups: Hispanic or Latino, non-Hispanic Black or African American, or non-Hispanic White. Individuals who were not classified as either Hispanic or Latino or non-Hispanic were included in the overall analysis but were excluded from subgroup analyses because their data were automatically censored when fewer than 10 deaths were recorded.

Urbanization was defined in accordance with the 2013 National Center for Health Statistics Urban-Rural Classification Scheme for Counties.^14^ Counties were classified as metropolitan or nonmetropolitan. Metropolitan counties were further subdivided, in decreasing order of urbanization, into large central metros (population over 1 million and containing the largest city or at least 250 000 residents within the statistical area), large fringe metros (population over 1 million but not central metro), medium metros (population between 250 000 and 999 999), and small metros (population less than 250 000). Nonmetropolitan counties were divided into micropolitan areas (urban clusters with populations between 10 000 and 49 999) and noncore areas (urban cluster with population less than 10 000).

The location of death was categorized into inpatient facility, nursing home, home, hospice facility, or other/unknown. The “other/unknown” category included locations that accounted for less than 5% of SDH-related deaths during the study period and encompassed unknown places of death and other locations not specified in the database. The hospice facility category was only available starting in 2003; before that, deaths were included in the other/unknown category.

SDH-related age-adjusted mortality rates (AAMRs) per 100 000 people were calculated by standardizing the crude mortality rate (CMR) to the 2000 US population as previously described.^15^ Both overall and subgroup segmented regression analyses were performed using the Joinpoint Regression Program (v4.9.1.0; National Cancer Institute, 2022).^16^ The year was used as the independent variable and annual percent change (APC) in mortality—approximated by the logarithm of AAMR—served as the dependent variable. This statistical method partitions the time series data into segments, each represented by its own linear regression model, to identify the specific years (Joinpoints) where significant changes in mortality rates occurred.

## RESULTS

A total of 203 295 SDH-related deaths occurred in older adults between 1999 and 2020 (AAMR 22.1 per 100 000 individuals). Of the total deaths, 110 684 (54.4%) were men, 92 611 (45.6%) were women, 170 953 (84.1%) were non-Hispanic White, 14 654 (7.2%) were non-Hispanic Black or African American, and 9656 (4.7%) were Hispanic or Latino. Of the 196 707 known locations of death, 134 397 (66.1%) were inpatient facilities, 30 863 (15.2%) were nursing homes, 15 960 (7.9%) were the decedent's home, and 15 960 (7.9%) were hospice facilities. Among diagnosis, age, sex, race and ethnicity, and urbanization, the highest mortality rates were seen in traumatic SDH (AAMR 13.7), those aged 85 years or older (CMR 73.6), men (AAMR 31.2), non-Hispanic White adults (AAMR 22.7), and micropolitan populations (AAMR 25.3) (Table).

**TABLE.** Demographics of subdural hemorrhage-related mortality from 1999 to 2020

Characteristics	Deaths (%)^a^	AAMR [95% CI]
Total	203 295 (100)	22.1 [22.0, 22.2]
Diagnosis		AAMR
Traumatic (S06.5)	126 343 (0)	13.7 [13.7, 13.8]
Nontraumatic (I62.0)	76 952 (0)	8.4 [8.3, 8.5]
10-y age groups		CMR
65-74 y	37 648 (18.5)	7.4 [7.3, 7.4]
75-84 y	77 581 (38.2)	26.0 [25.8, 26.2]
85+ y	88 066 (43.3)	73.6 [73.1, 74.1]
Sex		AAMR
Men	110 684 (54.4)	31.2 [31.0, 31.4]
Women	92 611 (45.6)	16.4 [16.3, 16.6]
Race/ethnicity		AAMR
Non-Hispanic White	170 953 (84.1)	22.7 [22.6, 22.8]
iNon-Hispanic Black or African American	14 654 (7.2)	19.7 [19.4, 20.0]
Hispanic or Latino	9656 (4.7)	16.5 [16.2, 16.8]
2013 urbanization		AAMR
Noncore	17 192 (8.5)	23.4 [23.0, 23.7]
Micropolitan	23 261 (11.4)	25.3 [24.9, 25.6]
Small metro	22 963 (11.3)	25.0 [24.7, 25.3]
Medium metro	46 069 (22.7)	23.4 [23.2, 23.6]
Large fringe metro	45 849 (22.6)	21.2 [21.0, 21.4]
Large central metro	47 961 (23.6)	19.2 [19.0, 19.4]
Census region		AAMR
Northeast	36 371 (17.9)	19.5 [19.3, 19.7]
Midwest	48 586 (23.9)	23.0 [22.8, 23.2]
South	76 044 (37.4)	23.2 [23.0, 23.4]
West	42 128 (20.7)	21.8 [21.5, 22,0]
Unknown	166 (0.1)	-
Location of death		
Inpatient	134 397 (66.1)	
Nursing home	30 863 (15.2)	
Home	15 487 (7.6)	
Hospice facility	15 960 (7.9)	
Other/unknown	6588 (3.2)	

AAMR, age-adjusted mortality rate per 100 000 people; CMR, crude mortality rate per 100 000 people.

a Percentages may not sum to 100 due to rounding

**FIGURE 1.**
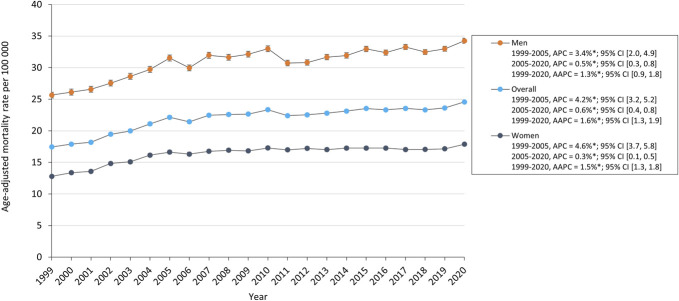
Trends in SDH-related age-adjusted mortality rates in older adults stratified by sex. AAPC, average annual percent change; APC, annual percent change; SDH, subdural hemorrhage.

**FIGURE 2.**
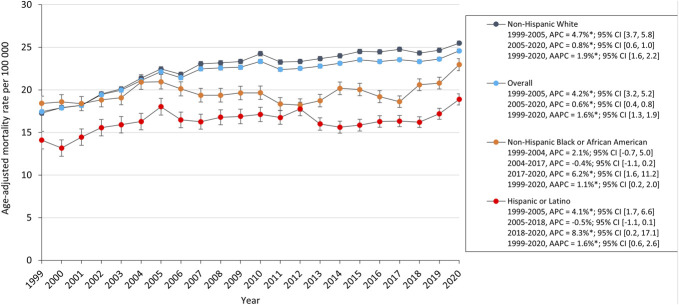
Trends in SDH-related age-adjusted mortality rates in older adults stratified by race and ethnicity. AAPC, average annual percent change; APC, annual percent change; SDH, subdural hemorrhage.

The overall AAMR for SDH in older adults increased from 17.4 in 1999 to 24.6 in 2020 with an average annual percent change of 1.3%; 95% CI (0.9, 1.8). The AAMR had an increase from 1999 to 2005 (APC, 3.4%; 95% CI [2.0, 4.9]) followed by a slower increase from 2005 to 2020 (APC, 0.5%; 95% CI [2.0, 4.9]). SDH-related mortality increased for both men and women. AAMR for men increased from 25.7 in 1999 to 34.3 in 2020, whereas for women increased from 12.8 in 1999 to 17.9 in 2020 (Figure 1). AAMR increased for all ethnic and racial groups. White adults had the highest AAMR which increased from 17.3 in 1999 to 24.5 in 2020, and Black and Hispanic groups increased from 18.4 and 14.1 to 23.0 and 18.9, respectively. Black and Hispanic adults demonstrated a period of stability from 2004 to 2017 (APC, −0.4% 95% CI [−1.1, 0.2]) and from 2005 to 2018 (APC, −0.5% 95% CI [−1.1, 0.1]), respectively, followed by a significant increase in AAMR to 2020 (Figure 2).

**FIGURE 3.**
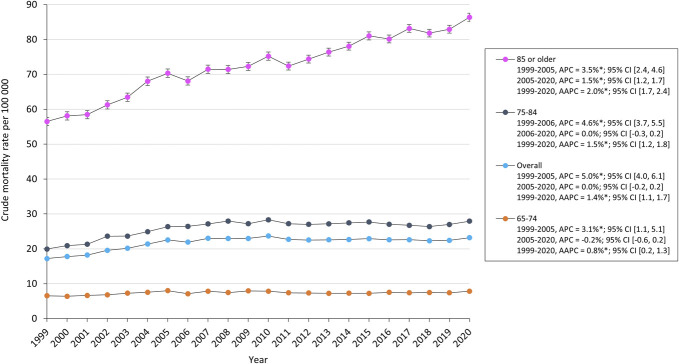
Trends in SDH-related crude mortality rates in older adults stratified by 10-year age group. AAPC, average annual percent change; APC, annual percent change; SDH, subdural hemorrhage.

Subgroup analysis showed that the combined CMR for older adults (65-74 years old, 75-84 years old, and 85 or older) increased from 17.2 in 1999 to 23.2 in 2020. When stratified by 10-year age groups, the CMR for adults aged 65 to 74 and 75 to 84 years increased from 1999 through 2005 and 2006, respectively, before stabilizing through 2020. However, the CMR in adults aged 85 years or older increased from 56.5 in 1999 to 86.4 in 2020 (Figure 3). From 1999 through 2020, the proportions of SDH-related deaths decreased in inpatient facilities (74.7%-56.5%) and nursing homes (19.3%-12.7%). By contrast, the proportion of SDH-related deaths increased in homes (4.1%-14.0%) and hospice facilities (0.2% in 2003 to 13.7% in 2020) (**Supplemental Digital Content 1** [http://links.lww.com/NS9/A55]).

**FIGURE 4.**
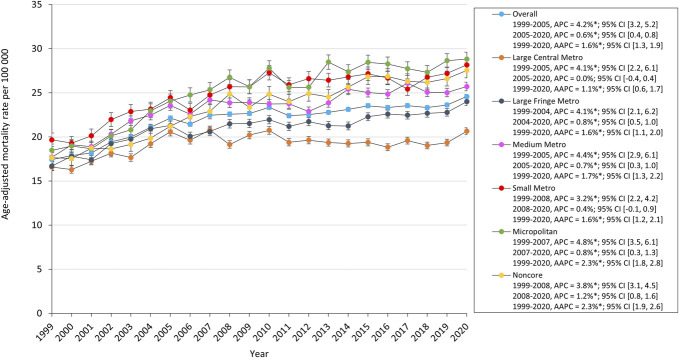
Trends in SDH-related age-adjusted mortality rates in older adults stratified by urbanization. AAPC, average annual percent change; APC, annual percent change; SDH, subdural hemorrhage.

**FIGURE 5.**
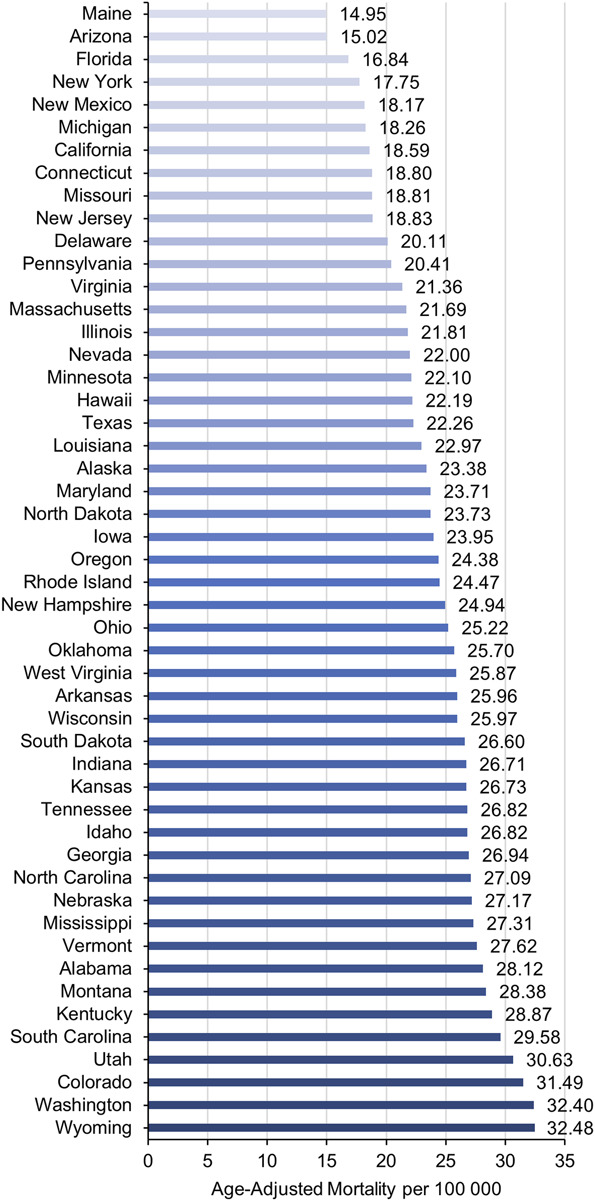
Overall SDH-related age-adjusted mortality rates in older adults stratified by state. AAPC, average annual percent change; APC, annual percent change; SDH, subdural hemorrhage.

The AAMR for all urbanization groups increased from 1999 to 2020. Micropolitan areas had the highest AAMR which increased from 18.5 in 1999 to 28.8 in 2020. Large central and small metropolitan areas increased from 1999 to 2005 and 2008, respectively, before stabilizing through 2020 (Figure 4). There were significant regional differences in the overall burden of SDH-related mortality with the Southern and Midwestern United States having the significantly higher mortality burden with an AAMR of 23.2 and 23.0, respectively, compared with the remaining census regions (Table). The top 5 states (Washington, Wyoming, Colorado, Utah, and South Carolina) with the highest SDH-related mortality had much higher mortality than those in the bottom 5 states (New Mexico, New York, Florida, Arizona, and Maine) (Figure 5).

## DISCUSSION

This 21-year analysis of mortality data for SDH patients aged 65 years or older reveals an overall increase in mortality from 1999 to 2020 with an overall AAMR of 22.1 per 100 000 individuals. The highest mortality rates occurred in cases of traumatic SDH, among patients aged 85 years or older, in non-Hispanic White adults, and within micropolitan populations. Notably, mortality rose significantly between 1999 and 2005, followed by a slower increase from 2005 to 2020. This escalating mortality, coupled with the rising incidence of SDH in the elderly, underscores a critical need for further research into the factors affecting this high-risk group, which is expected to comprise a growing portion of the population.

SDH is a common and possible catastrophic intracranial pathology, which refers to bleeding into a potential space between the dura and arachnoid mater. Approximately 11% and 20% of patients with significant head injuries develop an acute SDH.^4,17^ In one study involving 1150 patients with severe head injuries, 101 patients experienced an acute SDH. Only 19% of the cohort recovered functionally, with an overall mortality rate of 66%. Risk factors associated with the outcome included admission Glasgow Coma Scale score of 3 or 4, motorcycle accidents as a mechanism of injury, and age above 65 years.^18^ Elderly patients are at increased risk of SDH secondary to increased anticoagulant use, comorbidities, and cerebral atrophy.^1,4,10,11^ Cerebral atrophy in older individuals increases subdural space and places bridging veins under elevated tension, leading to increased risk of vascular injury. The incidence of SDH is increasing and is expected to rise in tandem with the expanding elderly population.^3-5,7,19^

Mortality associated with acute and chronic SDH likewise has been shown to increase with advancing age.^2,10,20^ For example, a study examining age-related mortality in 455 SDH patients found that age significantly influences both short-term and long-term survival after acute traumatic SDH. Older patients had substantially lower survival rates for several years postdischarge and experienced higher in-hospital mortality rates than adults with comparable injuries when compared with age-matched controls.^21^ However, there remains a scarcity of research detailing the long-term trends in SDH-related mortality rates in older adults. Studies that assess outcomes based on age often aggregate all individuals over 60 or 65 years together, practice that may obscure important variations within this patient cohort. Dumont et al found that increasing age at presentation is correlated with higher mortality from chronic SDH, particularly in patients aged older than 85 years.^10^ Our subgroup analysis demonstrated that the CMR for adults aged 65 to 74 and 75 to 84 years increased from 1999 to 2005 and 2006, respectively, before stabilizing through 2020. By contrast, the CMR for adults aged 85 years or older increased from 56.5 in 1999 to 86.4 in 2020. The population of individuals aged older than 85 years is projected to increase 115% by 2040.^7^ Therefore, developing effective risk-stratification measures is essential to ensure timely interventions and mitigate adverse outcomes in this vulnerable group.

Our analysis also indicated that while the SDH-related mortality rate in adults aged 65 years or older increased from 1999 to 2020, the rate of increase slowed after 2005. This slower rise may be due to improvements in surgical and medical management, as older patients are more likely to have comorbid conditions that receive treatment that may increase the risk of SDH. Unfortunately, the mortality rate continues to rise, and there is currently a paucity of evidence-based guidelines pertaining to surgical evacuation of SDH.^17^ The Surgical Management of Traumatic Brain Injury Author Group published recommendations for acute SDH in 2006 but no current guidelines for chronic SDH management.^17,20^ Common surgical evacuation techniques include craniotomy, craniectomy, and burr hole trephination,^20^ with SDH recurrence rates estimated between 2% and 37% after evacuation.^22^ Middle meningeal artery embolization (MMAE) has gained attention as an alternative treatment of chronic SDH. Srivatsan et al^23^ found that the recurrence rate of SDH after MMAE was significantly lower compared with conventional treatment; however, they did not analyze mortality rates. Although the popularity of MMAE is increasing, its impact is unlikely to be reflected in this analysis. Although outcomes of SDH evacuation are generally favorable,^17^ improvements in guidelines, surgical technique, and medical management are needed to combat the rising mortality in elderly patients.

### Sex

SDH-related mortality for both men and women increased from 1999 to 2020. Men had higher mortality rates at the beginning and end of the 21-year interval. However, the AAMR rose by 39.8% for women and 33.4% for men during this time. The larger increase in mortality rate among women may result from the higher percentage of older women in the growing elderly population. Currently, studies comparing SDH-related clinical outcomes and mortality rates between sexes are scarce and often yield inconsistent results.^24^

As for SDH incidence, a male predominance in SDH is well established. A retrospective cohort study examining prospective data from a trauma registry in Washington State between 2005 and 2008 reported that 63% of the 1427 SDH patients were male.^25^ Although this male bias in SDH formation is widely documented, the underlying cause remains unclear but are likely multifactorial. Marshman et al^26^ investigated potential risk factors contributing to the male bias in SDH in a study involving 155 cases, confirming the male predominance in SDH. Cerebral atrophy was identified as the main risk factor of chronic SDH, with similar rates in men and women (94% vs 91%). The established male bias was not explained by any known risk factor for chronic SDH development; of note, risk factor trends tended to favor women. Contrary to common assumptions, a male bias in chronic SDH was not associated with higher rates of trauma and/or alcohol misuse among men.

Collectively, the established male bias in SDH incidence and higher rate of male SDH-related mortality highlight an alarming sex-based disparity in SDH outcomes, as does the greater increase in AAMR among the female cohort. Additional research is crucial to elucidate the relationship between sex and SDH-related clinical outcomes and mortality to develop sex-specific interventions and guidelines.

### Race

All racial and ethnic groups experienced an increase in SDH-related mortality during the study period. Non-Hispanic African American adults had the greatest initial mortality rate with AAMR rising by 41.6%. Hispanic group had the second-highest starting morality rate with AAMR increase by 34%. There was a significant rise in the AAMR for African American and Hispanic adults until 2020 with periods of stability from 2004 to 2017 and from 2005 to 2018, respectively.

In a study examining traumatic brain injury and SDH patients between 2001 and 2004, 357 patients were categorized as White (46.2%), Asian (19.9%), Hispanic (17.9%), Black (10.9%), and other/unknown (5.0%). The study found that Asians had higher mortality rates than Whites but did not exhibit higher levels of disability. Hispanics seemed to fare slightly better after discharge when controlled for age and Injury Severity Score. A tendency for greater mortality among Asians and African American adults persisted.^27^

### Location and Other

Micropolitan regions exhibited the highest initial mortality rate from SDH, increasing by 55.6% over the study period. From 1999 to 2020, the AAMR increased across all urbanization groups. The number of major core and peripheral metropolitan areas increased between 1999 and 2008 and then stabilized until 2020. Each census area experienced a different overall burden of SDH-related mortality, with the US Midwest and South bearing the highest death burdens compared with other regions. Wyoming, Washington, Colorado, Utah, and South Carolina had the highest SDH-related mortality rates, whereas New Mexico, New York, Florida, Arizona, and Maine had the lowest overall AAMR.

### Limitations

This study has inherent limitations. Use ICD codes and death certificates may have misclassification errors of SDH as either a contributing or underlying cause of death. In addition, there may be coding errors or shifts in coding practices over time. Furthermore, the CDC WONDER database lacks detailed information regarding clinical course and management such as patient imaging, vitals, or laboratory results. The increased adoption of electronic medical records may lead to more frequent recording of SDH on death certificates, regardless of actual changes in SDH-related mortality. Therefore, the results of this study should be interpreted cautiously considering these inherent restraints.

## CONCLUSION

The study indicates that SDH-related mortality among older adults in the United States increased from 1999 to 2020. The AAMR of SDH-related mortality rose universally, albeit at varying rates across different demographic variables including age, sex, race and ethnicity, and geographic location. To reduce the growing burden of SDH-related mortality in the elderly population, it is imperative to implement targeted health policy measures focusing on identifying at-risk groups, coordinating effective interventions, and addressing existing disparities.

## Supplemental Digital Content

**Supplemental Digital Content 1**. **Figure.** Trends in SDH-related deaths in older adults stratified by location.
